# Efficient CO_2_ Electrocarboxylation Using Dye-Sensitized Photovoltaics

**DOI:** 10.3390/molecules30010040

**Published:** 2024-12-26

**Authors:** Yingtian Zhang, Huaiyan Ren, Huawei Zhou, Peipei Luo, Qi Wan, Xianxi Zhang, Bo Wang, Baoli Chen, Bo Zhang

**Affiliations:** School of Chemistry and Chemical Engineering, Liaocheng University, Liaocheng 252059, China; lcuytz@126.com (Y.Z.); rhy17862548687@163.com (H.R.); zhouhuawei@lcu.edu.cn (H.Z.); luopeipeigood@163.com (P.L.); w15689397767@163.com (Q.W.); xxzhang3@126.com (X.Z.); wangbo@lcu.edu.cn (B.W.)

**Keywords:** electrochemical carboxylation, electrocarboxylation, CO_2_ fixation, CO_2_ electrocatalytic carboxylation, electrosynthesis, dye-sensitized solar cells

## Abstract

This paper presents the solar-driven electrocarboxylation of 2-bromopyridine (2-BP) with CO_2_ into high-value-added chemicals 2-picolinic acid (2-PA) using dye-sensitized photovoltaics under simulated sunlight. Using three series-connected photovoltaic modules and an Ag electrode with excellent catalytic performance, a Faraday efficiency (*FE*) of 33.3% is obtained for 2-PA under mild conditions. The experimental results show that photovoltaics-driven systems for electrocarboxylation conversion of CO_2_ with heterocyclic halide to afford value-added heterocyclic carboxylic acid are feasible and effective.

## 1. Introduction

The concentration of carbon dioxide (CO_2_) in the atmosphere has continuously increased since the “Industrial Revolution”. Currently, the concentration of CO_2_ is over 400 ppm, and it is expected to triple by 2040 [1]. The excessive emission of CO_2_ has led to a series of environmental problems, such as global warming, glacier melting, and rising sea levels. Therefore, converting and utilizing CO_2_ is an urgent need and challenge for today’s society [2,3]. Additionally, CO_2_ is not only one of the main greenhouse gases but also a non-toxic, rich, and renewable C1 resource. Besides, electrochemical technology uses electrons as redox agents. In the electrochemical process, the gain and loss of electrons achieve the reduction and oxidation of substances, avoiding the problem of using oxidants and reducing agents in traditional chemical reactions, making electrochemical technology green and mild [4,5]. Thus, converting CO_2_ by electrochemical to produce value-added fuels or chemicals is an attractive way to achieve a carbon-neutral energy cycle [2,6,7,8,9]. However, in the process of CO_2_ electrochemical conversion, there is still a need for additional electricity supply issues.

In addition, the world’s energy demand is increasing significantly because of population growth and industrial evolution [10]. To overcome the increasingly serious global energy problem, green, renewable, and clean energy is considered the most feasible solution [11]. Among the green, renewable, and clean energy, solar energy, as one of the oldest energy resources on earth, is eco-friendly, easily accessible, and highly efficient [12,13]. It is widely used in photovoltaic power generation [14], solar thermal utilization, and artificial photosynthesis [15,16,17]. Among them, artificial photosynthesis refers to producing high-value-added chemicals using sunlight, which requires capturing its energy in chemical bonds that are easy to store and transport, and therefore, solves these two key challenges in solar energy utilization [15,18]. In the environmental and energy crisis that is imminent now, developing new, green, renewable energy and constructing an environmentally friendly and efficient energy system are of great significance and are receiving increasing attention [15]. Artificial photosynthesis towards the conversion of CO_2_ is undoubtedly a promising strategy.

To this day, artificial photosynthesis is mainly towards sunlight-driven water splitting to produce O_2_ and H_2_ [17,19] and CO_2_ electroreduction to various high-value-added fuels [20], including carbon monoxide (CO) [15], methane [21], ethylene [22], and ethanol [23]. For instance, Schreier et al. [15] convert CO_2_ to CO by using perovskite photovoltaics solar cells connected in series to supply the required current and voltage for CO_2_ electroreduction. Asadi et al. [24] study the CO_2_ photosynthesis for CO_2_ electroreduction to CO using two amorphous silicon triple-junction photovoltaic cells connected in series to provide the required current and voltage for CO_2_ electroreduction. However, reports on photosynthesis directed toward the electrocarboxylation of organic molecules with CO_2_ are scarce, and related research is highly required [25,26,27,28]. Closely related to our investigation, lots of effort has been devoted to the investigation of the electrocarboxylation of numerous substrates with CO_2_, including alcohols [29,30], alkenes [31,32], organohalides [33,34,35], imines [36], and ketones [37,38] under mild conditions to obtain a list of high-value-added carboxylated chemicals.

Moreover, the emergence of dye-sensitized solar cells (DSCs) has aroused extensive attention in the photovoltaic community because they possess many advantages, such as being green and low-cost [39,40]. Inspired by the artificial photosynthesis of water to produce O_2_ and H_2_ and the artificial photosynthesis of the electroreduction of CO_2_ into fuels, we decided to extend our previous work [6,34,41,42] for carbon neutrality and energy utilization. In this article, we used DSCs as the photoreaction and CO_2_ conversion as the dark reaction, using a solar energy system to convert CO_2_ to value-added carboxylation product aromatic carboxylic acid. We demonstrate the efficient sunlight-driven photosynthesis for carboxylation of 2-bromopyridine (2-BP) with CO_2_ using dye-sensitized photovoltaics to produce the carboxylation product 2-picolinic acid (2-PA), which is an important organic synthetic intermediate used in the pharmaceutical industry for the preparation of capocaine drugs and neuro pharmaceuticals and is widely used in nerve suppression and local anesthesia. Using dye-sensitized photovoltaics connected in series and an Ag electrode with excellent catalytic performance, we achieved a 30.3% Faraday efficiency (*FE*) for 2-PA under mild conditions.

## 2. Results and Discussion

**Figure 1:**
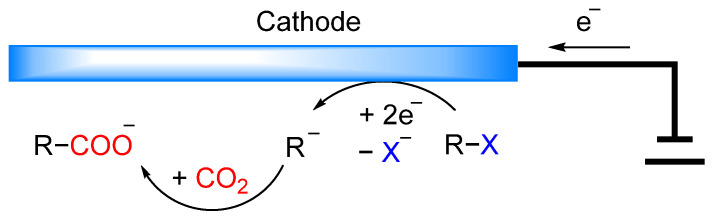
The general mechanism for electrocarboxylation of organohalides (RX) in the presence of CO_2_.

Electrocarboxylation of organohalides with CO_2_ is a direct and effective way to construct value-added aromatic carboxylic acids and their derivatives [43,44]. In general, the organohalides would preferentially undergo reduction at the cathode [43] when the reduction potential for CO_2_ is lower than that for organohalides. The general electrocarboxylation mechanism of organohalides (RX) in the presence of CO_2_ includes a two-electron reduction of RX to R^−^, subsequently followed by the nucleophilic attacking CO_2_ of R^−^ (as shown in Figure 1) [41,43,45]. Moreover, research has shown that the Ag electrode exhibits excellent catalytic activity in the electrocarboxylation of organohalides with CO_2_, making a positive shift of the reduction potential values for the fracture of the C–X bond in organohalides [35,38], and is therefore often selected as a working electrode. In addition, 2-BP is one of the simplest N-heterocyclic aromatic halides, and in this study, the electrocarboxylation of 2-BP in the presence of CO_2_ was selected for the investigation of artificial photosynthesis using dye-sensitized photovoltaics connected in series and an Ag electrode with excellent catalytic performance.

### 2.1. Cyclic Voltammetry (CV) Behavior for 2-BP and Controlled-Potential Electrolysis of 2-BP with CO_2_

**Figure 2:**
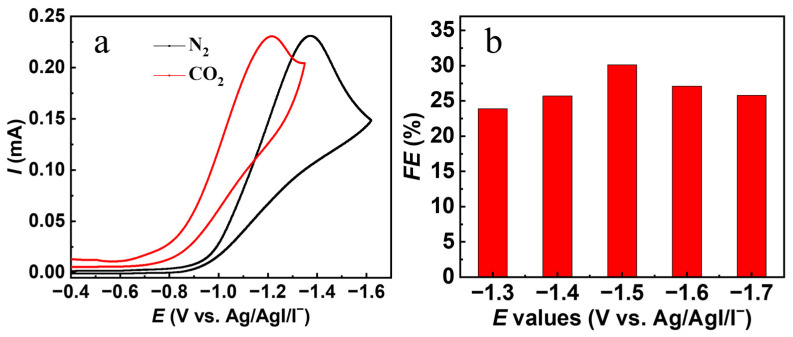
(**a**) The cyclic voltammograms of 2-BP (10 mM) at an Ag electrode in the absence (black) and presence (red) of CO_2_; (**b**) the *FE* of carboxylation product 2-PA with different *E* values.

CV studies of 2-BP were first carried out in a three-electrode system (Figure 2a). Considering that the Ag electrode exhibits excellent catalytic activity towards the electroreduction of organic halides in the presence of CO_2_ (i.e., electrocarboxylation of organic halides), the Ag electrode (d = 2 mm) was chosen as the cathode that is the working electrode [41,43]. The counter electrode, that is, the anode, is a platinum (Pt) wire, the reference electrode is the Ag/AgI/0.1 M tetrabutylammonium iodide (TBAI) in DMF, the solvent is DMF, and the supporting electrolyte is tetraethylammonium tetrafluoroborate (TEABF_4_). The scanning direction for the CV study was first negative scanning (i.e., scanning from high potential to low potential), followed by positive scanning. The black line of Figure 2a is the CV behavior of 2-BP in the presence of N_2,_ with a potential window ranging from −0.40 to −1.62. (vs. Ag/AgI/I^−^). As the black line in Figure 2 shows, a single irreversible peak was observed during negative scanning with the potential of −1.37 V (vs. Ag/AgI/I^−^), which represents the occurrence of two-electron reduction of 2-BP to the corresponding dehalogenated carbanion [41,46]. In addition, the reduction current begins to rise at the potential of about −0.80 V (vs. Ag/AgI/I^−^). The introduction of CO_2_ significantly alters the voltammetric behavior. As we can see from the red line of Figure 2a, after DMF was saturated with CO_2_, the remarkable positive shift for the reduction peak potential of 2-BP from −1.37 to −1.22 V (vs. Ag/AgI/I^−^) was displayed during negative scanning. Meanwhile, the reduction current began to rise at the potential of about −0.60 V (vs. Ag/AgI/I^−^). These results imply that the presence of CO_2_ is favorable for the fracture of the C-Br bond of 2-BP and the existence of a fast reaction between the electrogenerated carbanion of 2-BP and CO_2_ (Figure 1) [41,46].

**Scheme 1:**
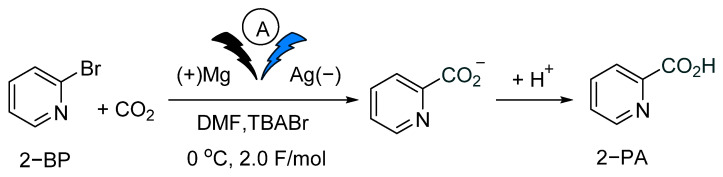
Carboxylation of 2-BP with CO_2_ to gain the corresponding carboxylation products 2-picolinic acid (2-PA), the letter A represents current.

Next, the controlled-potential electrolysis experiment in a CO_2_-saturated DMF solution including 0.1 M 2-BP was conducted using a three-electrode system (a ring Ag (S = 8 cm^2^) cathode, a sacrificial Mg anode, and an Ag/AgI/0.1 mol/L TBAl reference electrode) at different electrolytic potentials till it passed through 2 F/mol of charge at 0 °C. Based on our previous work, the CV behavior of 2-BP above, and literature reports, it is known that the electroreduction of 2-BP with CO_2_ undergoes a two-electron reduction process (involving the fracture of the C–X bond in 2-BP), producing the corresponding dehalogenated carbanion, which nucleophilic attacks CO_2_ and ultimately generates the carboxylic acid 2-picolinic acid (2-PA) after hydrolyzed (Scheme 1) [41,43,47]. After electrolysis and hydrolysis, the target carboxylic acid was thus obtained, characterized by high-resolution mass spectrometry (HRMS), and detected by high-performance liquid chromatography (HPLC). HRMS analysis (2-PA, HRMS (ESI) *m*/*z* [M + H]^+^ calculated for C_6_H_6_NO_2_ 124.0399 found 124.0400) showed that 2-PA was obtained as the electrocarboxylation product (the molecular formula is shown in Scheme 1). Moreover, by comparing the HPLC peak time of the target product for electrocarboxylation of 2-BP with that of a commercially available 2-PA (99% purity), it was also indicated that the 2-PA was obtained. To test the Faraday efficiency (*FE*) for carboxylation product 2-PA, the concentration of 2-PA was measured by HPLC with the help of the standard curve (Figure S1). Then, the *FE* of 2-PA was obtained using the following formula:

(1) $$FE=\frac{n_{2-PA}\times z\times F}{Q}\times 100\%$$

In Equation (1), *z* is the number of electron transference during the electroreduction of 2-BP to 2-PA, which equals 2, *n* is the real detected number of moles of the target product 2-PA (shown in Scheme 1), *F* is the Faraday constant, and *Q* is the amount for the charge passed during the electrolysis process, which equals to 193 C here. Figure 2b shows the *FE* of carboxylation product 2-PA when potential with different *E* values were applied to the electrocarboxylation of 2-BP with CO_2_. As shown in Figure 2b, the *FE*s of 2-PA at different *E* values −1.3, −1.4, −1.5, −1.6, and −1.7 V are 23.9%, 25.7%, 30.1%, 27.1%, and 25.8%, respectively. The highest *FE* is 30.1% at −1.5 V.

### 2.2. Artificial Photosynthesis for Electrocarboxylation of 2-BP with CO_2_ Using Dye-Sensitized Photovoltaics

**Figure 3:**
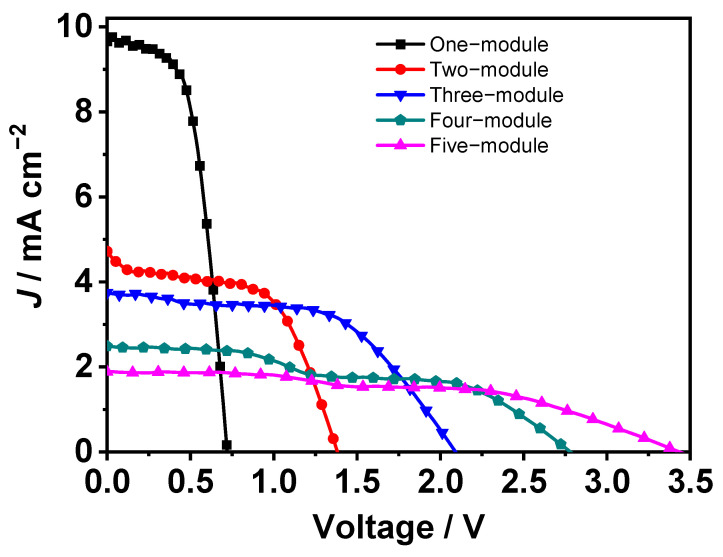
The current density-voltage (J-V) curve of the DSCs module under AM1.5 simulated sunlight.

Firstly, the preparation of dye-sensitized solar cells (DSCs) was conducted, and the specific preparation process was shown in the section on materials and methods. The DSCs consist of an electrolyte, a cathode, and a photoanode. The photoanode is a dye (N719)-sensitized TiO_2_ mesoporous film (12 μm). The electrolyte composition is as follows: 0.1 M LiI, 0.03 M I_2_, 0.6 M 1-butyl-3-methyl-imidazolium iodide (BMII), 0.5 M (4-tert-butylpyridine) TBP, 0.1 M (guanidinium thiocyanate) GuSCN, and the solvent is acetonitrile. The cathode electrode is pyrolysis Pt. The photoanode and cathode are encapsulated through polymer film (45 μm). The aluminum foil acted as an electronic collection line between each DSC in the photovoltaic model. Moreover, the DSCs were series-connected to obtain different potentials. Figure 3 exhibits the current density-voltage (J-V) curves for the DSCs model under AM1.5 simulated sunlight (100 mW cm^−2^). As presented in Figure 3, the photoelectric parameters of a single DSC are short circuit current density (J_sc_) = 9.65 mA cm^−2^, open circuit voltage (V_oc_) = 0.72 V, fill factor (FF) = 59.07%, and power conversion efficiency (PCE) = 4.11%. The photoelectric parameters of two DSCs with series connection are J_sc_ = 4.77 mA cm^−2^, V_oc_ = 1.38 V, FF = 53.48%, and PCE = 3.53%. The photoelectric parameters for three DSCs with series connection are J_sc_ = 3.75 mA cm^−2^, V_oc_ = 2.09 V, FF = 56.22%, and PCE = 4.41%. The photoelectric parameters of four DSCs with series connection are J_sc_ = 2.48 mA cm^−2^, V_oc_ = 2.78 V, FF = 49.05%, and PCE = 3.38%. The photoelectric parameters of five DSCs with series connection are J_sc_ = 1.85 mA cm^−2^, V_oc_ = 3.44 V, FF = 51.72%, and PCE = 3.29%. Typically, the PCE of DSCs with a small area (the classic area is 0.16 cm^2^) could reach 7–8% [48,49]. Figure 3 shows the PCE of the DSCs here is generally not high (up to 4.41%), mainly due to the large area of the DSCs. When the DSCs with series connection are one, two, three, four, and five, the area of the DSCs is 2.75, 5.50, 8.25, 11.00, and 13.75 cm^2^, respectively. Generally, when the area of the DSCs increases, the block resistance of the conductive glass increases, resulting in lower PCE. In addition, Figure 3 displays that the current density of the series-connected module significantly decreases as the number of series DSCs increases. This is because as the number of DSCs connected in series increases, the wires and connecting materials of the series module will become longer, resulting in a rise in the resistance of the series module, ultimately leading to a decline in short-circuit current or current density.

**Figure 4:**
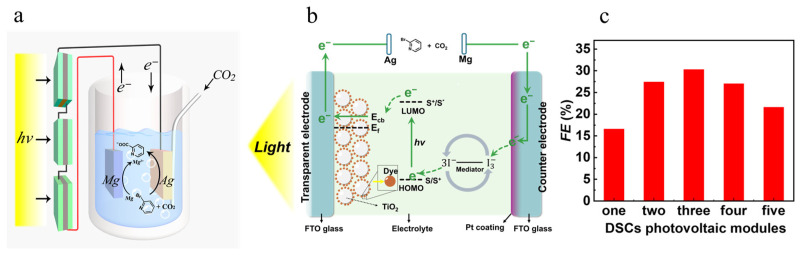
(**a**) The detailed diagram of electrocarboxylation of 2-BP with CO_2_ using dye-sensitized photovoltaics; (**b**) the diagram of energy and electron transfer for the photoreaction; (**c**) the *FE* of electrocarboxylation of 2-BP with CO_2_ into 2-PA using different quantities of DSCs connected in series.

Afterward, the series-connected DSCs were used as the power under only solar light to activate the carboxylation reactions of 2-BP in artificial photosynthesis. To obtain the carboxylation product in artificial photosynthesis, the DSCs as the photoreaction are linked to the carboxylation reactions of 2-BP according to Figure 4a. The composition of the carboxylation reaction is as follows: the cathode is an Ag sheet (S = 8 cm^2^), the anode is a Mg rod, and the solvent is 10 mL DMF with 0.1 M 2-BP. In the experiment, the gas CO_2_ was passed into the reaction solution close to the Ag electrode. The diagram of energy and electron transfer for the photoreaction is shown in Figure 4b, and the specific artificial photosynthesis process is as follows: Sunlight was irradiated to the photoanode of the DSCs and absorbed by N719. The electrons in N719 transited from the ground state to the excited state, and then the electrons in an excited state were infused into the conduction band of the semiconductor (TiO_2_) and were collected by FTO. The electrons in the external circuit reached the Ag sheet. With the catalysis of Ag [41,50,51], 2-BP underwent a two-electron transfer at the Ag cathode, generating dehalogenated carbanion, which then nucleophilic attacked CO_2_ to produce corresponding carboxylation product 2-PA after work-up (Scheme 1) [41,43,45,47]. CO_2_ gas was continuously injected into the system under a slow stream at normal pressures, and the reaction continuously operated till it passed through 2 F/mol of charge at 0 °C, and simultaneously, the simulated sunlight was switched off. In addition, the Mg anode underwent an oxidation reaction and was connected to the cathode of DSCs. The I_3_^−^ ions produced by oxidation of I^−^ diffused through the electrolyte solution to the platinum catalyst cathode and regenerated I^−^. The dye was restored to its ground state by electron transfer from I^−^ in iodide/triiodide electrolyte. Then, the electron transport process of the photoreaction and dark reaction was completed. In addition, to calculate the *FE* of the carboxylation product, the total amount of the passed *Q* needs to be calculated, and the method is as follows: The UT61E multimeter communicated with the office computer using an RS232 serial port transmitted the recorded current value for the reaction system to the office computer per 0.1s, and then the information was leading-in into Microsoft Office Excel 2016 to count the real-time *Q* passed.

After this artificial photosynthesis, the product was hydrolyzed, and the target carboxylic acid was thus obtained, characterized by HRMS, and detected by HPLC. HRMS analysis showed that 2-PA (the structure shown in Scheme 1) was obtained as the carboxylation product of the electrocarboxylation of 2-BP with CO_2_ using photovoltaics. Moreover, by comparing the HPLC peak time of the target product with that of a high-purity (99% purity) commercially available 2-PA, it was also indicated that 2-PA was obtained. Also, to test the carboxylation *FE* of 2-PA, the concentration of 2-PA was measured by HPLC with the standard curve (Figure S1), and then, the *FE* of 2-PA was obtained using the Formula (1). As shown in Figure 4c, the *FE*s of 2-PA under one, two, three, four, and five DSCs with series connection are 16.6%, 27.4%, 30.3%, 27.0%, and 21.6%, respectively. The highest *FE* is 30.3%, obtained with three DSCs with series connection, almost equal to the highest *FE* (30.1%, Figure 2b) by the ordinary electrocarboxylation of BP with CO_2_. The results illustrate that the electrocarboxylation of 2-BP with CO_2_ using photovoltaics is feasible, and this system offers a promising pathway to convert CO_2_ into value-added chemicals under mild conditions by using clean energy while reducing the concentration of CO_2_.

**Figure 5:**
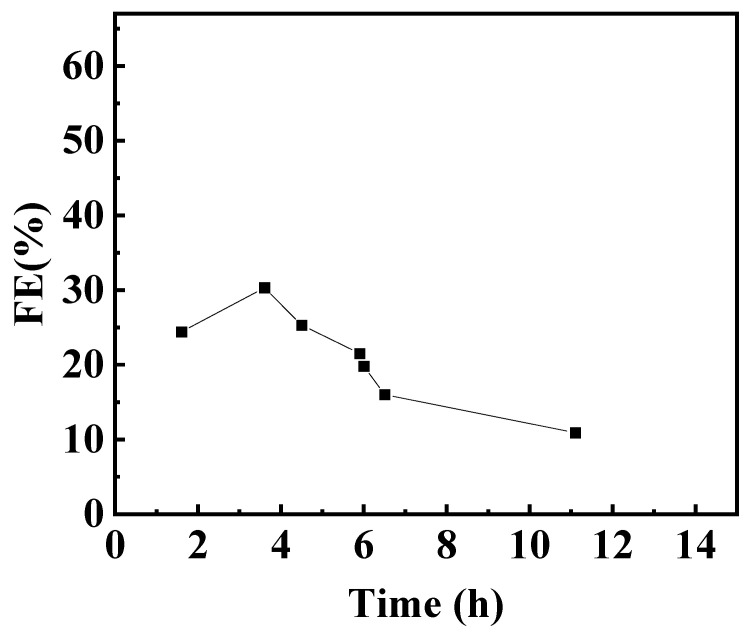
The stability of the electrocarboxylation of 2-BP with CO_2_ into 2-PA using dye-sensitized photovoltaics.

In addition, the stability of this artificial photosynthesis for electrocarboxylation of 2-BP with CO_2_ was also carried out with three series-connected photovoltaic modules. As displayed in Figure 5, the *FE* of 2-PA still maintains 83.5% of the best value for this artificial photosynthesis after 4.5 h, which indicates that artificial photosynthesis for electrocarboxylation of 2-BP with CO_2_ to 2-PA is effective.

## 3. Materials and Methods

### 3.1. Chemicals and Instruments

2-Bromopyridine (2-BP, purity of 99%) and 2-picolinic acid (2-PA, purity of 99%) were procured from Beijing J&K Technology Co., Ltd. (Beijing, China). Tetraethylammonium tetrafluoroborate (TEABF_4_, purity of 99%) was procured from Alfa Aesar Chemical Co., Ltd. (Shanghai, China). The other reagents were obtained from the Sinopharm Chemical Reagent. Co., Ltd. (Beijing, China). *N*,*N*-dimethylformamide (DMF) was dehydrated with 4 Å molecular sieves before use. All the other reagents (purities > 99%) were used directly without further processing. The high-resolution mass spectrometry (HRMS) was obtained on a Waters mass spectrometer (XEVO G2-XS QTOF, Waters, Milford, MA, USA) with ESI resources. The product concentrations were obtained through high-performance liquid chromatography (HPLC, Waters 505 pump, Waters, Milford, MA, USA), which was linked to a Waters 2489 UV detector (Waters, Milford, MA, USA) and a C18 column. Cyclic voltammograms and controlled-potential electrolysis were performed on a Chenhua CHI760E electrochemical station from Shanghai Chenhua Instruments Company (Shanghai, China). The electrocarboxylation using dye-sensitized photovoltaics was conducted using DSCs and a UNT-T UT61E multimeter (Ulide Technology (China) Co., Ltd., Dongguan City, China) connected to a Lenovo computer along with a CEL-S500-T5 solar simulator from Beijing China Education Au-light Co., Ltd. (Shanghai, China). The J-V curves of the DSCs were conducted by an IV5 IV testing system from PV Measurements, Inc. (Washington, DC, USA) with the help of a CEL-S500-T5 solar simulator from Beijing China Education Au-light Co., Ltd. (Shanghai, China).

### 3.2. A Typical Procedure for Cyclic Voltammogram and Controlled-Potential Electrolysis of 2-BP with CO_2_

The typical electroanalytical experiment was conducted in an undivided cell using a three-electrode system. The Ag disk (d = 2 mm) electrode was used as the working electrode, which is the cathode; the Pt wire was used as the auxiliary electrode, which is the anode here; and Ag/AgI/0.1 M TBAI in DMF was used as the reference electrode. The experimental electrolyte was prepared by 10 mM 2-BP and 0.1 M TEABF_4_ in 10 mL DMF solvent. All the experiments were carried out at room temperature under atmospheric pressure with a 0.1 V/s scanning rate. In addition, the typical controlled-potential electrolysis was conducted in CO_2_-saturated 10 mL DMF solution under atmospheric pressure, including 0.1 M supporting electrolyte tetrabutylammonium bromide (TBABr) and 0.1 M 2-BP in an undivided glass cell possessing a ring Ag (S = 8 cm^2^) cathode, a reference electrode Ag/AgI/0.1 M TBAl, and a sacrificial Mg rod anode at 0 °C, until charge (*Q*) of 2 F/mol was passed. After the electrolysis reaction was completed, the solution was distilled off, and the residual mixture was hydrolyzed in a mixture solution of a NaH_2_PO_4_/Na_2_HPO_4_ buffer at pH 6 and MeCN (76/24, *v*/*v*), then the target carboxylic acid (shown in Scheme 1) was thus obtained. Finally, the aimed product 2-PA was characterized by HRMS and detected by HPLC, and the HPLC peak time for the target carboxylic acid was compared with that of a commercially available 2-PA (99% purity). Meanwhile, the *FE* of the target carboxylation product 2-PA was evaluated by Equation (1) and HPLC with the calibration curves (Figure S1) after appropriate dilution. The eluent was a mixture solution of a NaH_2_PO_4_/Na_2_HPO_4_ buffer at pH 6 and MeCN (88/12, *v*/*v*), and the detection wavelength was 265 nm.

### 3.3. A Typical Procedure for the Preparation and Assembling of DSCs and the Artificial Photosynthesis for Electrocarboxylation of 2-BP with CO_2_ Using Dye-Sensitized Photovoltaics

A typical procedure for preparing and assembling DSCs is as follows: Step 1: spray the aqueous solution of H_2_PtCl_6_ (8% wt) onto the FTO conductive glass that has been cleaned thoroughly. Next, heat the H_2_PtCl_6_ on FTO at 80 °C on a hot plate, and then heat to 450 °C to obtain the counter electrode. Step 2: screen printing technology was used to print TiO_2_ (10 μm) paste onto FTO conductive glass to a thickness of 10 mm. Next, heat the TiO_2_ film at 500 °C for 60 min. The photoanode was treated with an aqueous solution of TiCl_4_ (0.04 M) for 30 min, according to reference [42]. Following, heat the film of TiO_2_ again at the temperature of 500 °C for 30 min. When the film was cooled down to 80 °C, immersed in the N719 dye (10^−5^ M) with ethanol as a solvent for 20 h to prepare the photoanode, and placed in a dark corner. Step 3: place a surlyn film on the photoanode according to the size of the electrode area. Subsequently, place another electrode, the Pt electrode, on the surlyn film to form a sandwich structure of photoanode/surlyn film/Pt electrode and stack the sandwich device together with the help of fixtures. Next, heat the sandwich device at the temperature of 180 °C for 3 min, then remove it from the hot plate and cool it to room temperature. Step 4: inject the electrolyte (0.5 mL) into the gap between two electrodes through a small hole, and vacuum extract the bubbles to evenly fill the electrolyte in the electrode gap. Next, UV glue is used to seal the small hole, and then a UV lamp is used to irradiate it for 10–20 min to cure the UV glue fully, completing the packaging of the sandwich device. Then, DSCs were series-connected (while their J-V curves were being tested) for subsequent use. In addition, the typical artificial photosynthesis was carried out using DSCs connected in series to replace CHI760E as the power and linked the DSCs module to dark reactions (carboxylation reactions) in Figure 4a. The series-connected DSCs were illuminated using AM1.5 simulated sunlight (100 mW cm^−2^). The carboxylation reactions were run in CO_2_-saturated 10 mL DMF solution, including 0.1 M 2-BP and 0.1 M supporting electrolyte TBABr in an undivided glass cell possesses a two-electrode system that a ring Ag (S = 8 cm^2^) cathode and a sacrificial Mg anode at 0 °C, until *Q* of 2 F/mol was passed under atmospheric pressure. After the artificial photosynthesis reaction, the solution was distilled off, and the residual mixture was hydrolyzed in a mixture solution of a NaH_2_PO_4_/Na_2_HPO_4_ buffer at pH 6 and MeCN (76/24, *v*/*v*), then the target carboxylic acid (shown in Scheme 1) was thus obtained. Finally, the aimed product 2-PA was characterized by HRMS and detected by HPLC, and the HPLC peak time for the target carboxylic acid was compared with that of a purchased high-purity 2-PA (99% purity). The *FE* of the carboxylation product 2-PA was evaluated by Equation (1) and HPLC with the help of the calibration curves (Figure S1) after appropriate dilution. The eluent was a mixture solution of a NaH_2_PO_4_/Na_2_HPO_4_ buffer at pH 6 and MeCN (88/12, *v*/*v*), and the detection wavelength was 265 nm.

## 4. Conclusions

In summary, an efficient artificial photosynthesis system was successfully constructed for electrocarboxylation of 2-BP with CO_2_ to afford the corresponding value-added carboxylation product N-heterocyclic carboxylic acid 2-PA under mild conditions. We obtained a 30.3% *FE* for 2-PA using three dye-sensitized photovoltaics connected in series and an Ag electrode with excellent catalytic performance. Moreover, stability experiments showed that the entire CO_2_ artificial photosynthesis system was effective. This study provides a new pathway for efficient CO_2_ electrocarboxylation with heterocyclic halide to generate high-value-added chemical heterocyclic carboxylic acid, and future work will focus on exploring more applications of dye-sensitized photovoltaics in CO_2_ electrocarboxylation to produce a greater diversity of value-added chemicals.

## Data Availability

Data are contained within this article and Supplementary Materials.

## Supplementary Materials

The following supporting information can be downloaded at: https://www.mdpi.com/article/10.3390/molecules30010040/s1, Figure S1: Standard curve of 2-picolinic acid for quantitative analysis.
