# Supplementary material for: Efficient CO2 Electrocarboxylation Using Dye-Sensitized Photovoltaics
Source: Molecules. 2024 Dec 26;30(1):40. doi: 10.3390/molecules30010040 (PMC11721401; doi:10.3390/molecules30010040)
Supplement: Supplementary file 1 [file molecules-30-00040-s001.zip › molecules-3380403-supplementary.pdf]

Supplementary Material for

Efficient CO_2 Electrocarboxylation Using Dye-Sensitized Photovoltaics

Yingtian Zhang, Huaiyan Ren, Huawei Zhou, Peipei Luo, Qi Wan, Xianxi Zhang, Bo Wang, Baoli Chen * and Bo Zhang *

School of Chemistry and Chemical Engineering, Liaocheng University; Liaocheng 252059, China.
*Corresponding Author: E-mail: chenbaoli@lcu.edu.cn (B.C.); bzhang@lcu.edu.cn (B.Z.)

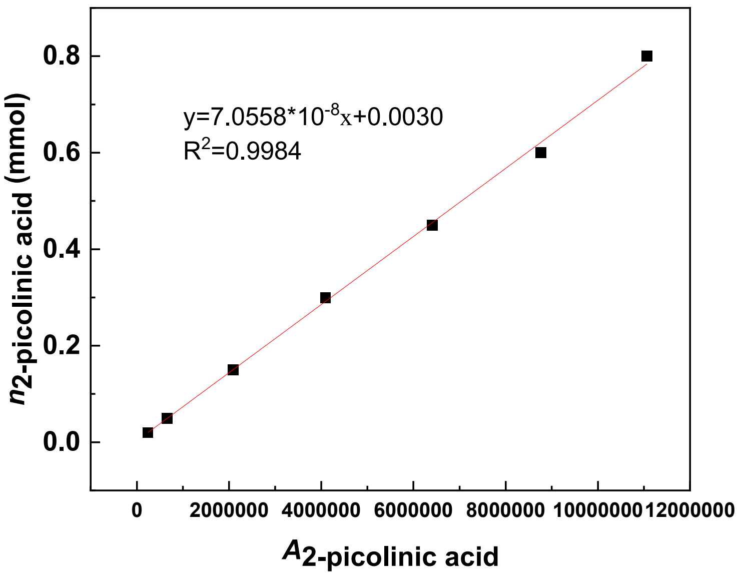

Figure S1. Standard curve of 2-picolinic acid for quantitative analysis.

The standard curve of the carboxylation product 2-picolinic acid is shown in Figure S1. The mathematical expression of the standard curve is y = 7.0558*10^{-8}x + 0.0030 (R^2 = 0.9984), and the linearity is good. Among them, the *y*-axis is the number of moles of 2-picolinic acid, and the *x*-axis is the peak area of 2-picolinic acid ($A_{\text{2-picolinic acid}}$). According to the standard curve equation, the number of moles of the target product 2-picolinic acid can be calculated.
